# Pharmacovigilance in the digital age: gaining insight from social media data

**DOI:** 10.3389/ebm.2025.10555

**Published:** 2025-05-27

**Authors:** Fan Dong, Wenjing Guo, Jie Liu, Tucker A. Patterson, Huixiao Hong

**Affiliations:** National Center for Toxicological Research, U.S. Food and Drug Administration, Jefferson, AR, United States

**Keywords:** drug safety, artificial intelligence, machine learning, natural language processing, social media, post-market surveillance

## Abstract

Pharmacovigilance is essential for protecting patient health by monitoring and managing medication-related risks. Traditional methods like spontaneous reporting systems and clinical trials are valuable for identifying adverse drug events, but face delays in data access. Social media platforms, with their real-time data, offer a novel avenue for pharmacovigilance by providing a wealth of user-generated content on medication usage, adverse drug events, and public sentiment. However, the unstructured nature of social media content presents challenges in data analysis, including variability and potential biases. Advanced techniques like natural language processing and machine learning are increasingly being employed to extract meaningful information from social media data, aiding in early adverse drug event detection and real-time medication safety monitoring. Ensuring data reliability and addressing ethical considerations are crucial in this context. This review examines the existing literature on the use of social media data for drug safety analysis, highlighting the platforms involved, methodologies applied, and research questions explored. It also discusses the challenges, limitations, and future directions of this emerging field, emphasizing the need for ethical principles, transparency, and interdisciplinary collaboration to maximize the potential of social media in enhancing pharmacovigilance efforts.

## Impact statement

Pharmacovigilance explores the transformative potential of social media in enhancing drug safety monitoring. Traditional methods, while foundational, are limited by delayed data collection and analysis, creating gaps in timely adverse drug event detection. This review advances the field by examining the latest methodologies, including natural language processing and machine learning that enable the extraction of meaningful information from unstructured social media data. These advanced techniques provide tools to overcome challenges such as data variability and bias, making social media a viable complement to established pharmacovigilance practices. The insight from this review impacts the field by demonstrating how social media can fill critical gaps in real-time adverse drug event detection and provide a broader understanding of public sentiment and patient experiences. By emphasizing the need for interdisciplinary collaboration, ethical principles, and transparency, this review lays the foundation for a more agile, inclusive, and effective pharmacovigilance system, ultimately enhancing public health outcomes.

## Introduction

Pharmacovigilance is an essential component of healthcare, focused on safeguarding patients by vigilantly monitoring and managing the risks associated with medications. Traditional pharmacovigilance methods, such as spontaneous reporting systems and clinical trial data analysis, have played pivotal roles in detecting and preventing adverse drug events. However, these methods have limitations, including delayed access to critical information due to the lag between data collection and availability. This lag impedes the timely monitoring of medication safety, thereby posing challenges to real-time surveillance.

The advent of social media has introduced a new paradigm in pharmacovigilance, providing a platform for individuals to share their experiences and opinions about medications. Platforms like X (former name Twitter) and Facebook serve as repositories of user-generated content, which offer discernment into medication usage patterns, adverse drug events, and public sentiment surrounding pharmaceuticals. Such a reservoir of data can complement traditional data sources by providing near real-time information on medication safety concerns.

Despite its potential, relying solely on social media data for pharmacovigilance has challenges. A significant issue arises from the unstructured nature of social media content, which can introduce variability and noise into the data, thereby complicating the identification of drug safety signals. Furthermore, social media users may not represent the entire population, leading to potential biases in demographic representation and geographic coverage.

To overcome these challenges, advanced analytical techniques such as natural language processing and machine learning are being employed to analyze social media data more effectively. These methods enable the identification of patterns and trends, thereby assisting in the early detection of potential safety concerns and facilitating real-time monitoring of medication safety. It is crucial to ensure the reliability of social media data by validating findings with conventional data sources and maintaining strict privacy and ethical standards in data usage.

The use of social media data in pharmacovigilance represents a promising strategy for enhancing medication safety monitoring. By addressing the accompanying challenges and employing rigorous methodologies, social media data can serve as a valuable complement to conventional surveillance data. In the future, prioritizing ethical principles, promoting transparency, and fostering interdisciplinary collaboration will prove indispensable in fully harnessing the potential of social media data in safeguarding public health.

Social media platforms have emerged as significant sources of real-time data, where users freely share their experiences and opinions on various topics, including healthcare and medication usage. These platforms enable the collection of patient-reported outcomes, capture discussions on medication usage and safety, and identify the dissemination of misinformation related to drugs and vaccines. Leveraging the capabilities of these platforms empowers researchers and healthcare professionals to delve into the experiences, attitudes, and concerns of the public. This wealth of user-generated content provides a unique perspective on drug safety and efficacy, offering insight that complements traditional pharmacovigilance methods and enhancing our understanding of medication impact in real-world settings.

The use of these platforms addresses a multitude of research questions, spanning from understanding the reasons behind medication changes to evaluating public sentiments towards pharmacovigilance endeavors, and even combating the spread of health-related misinformation. This review examines the current literature on the use of social media data for drug safety analysis, with a focus on the platforms utilized, the key information extracted, the research questions addressed, and the methodologies applied. Additionally, the review discusses the challenges, limitations, and future directions of utilizing social media data for drug safety analysis.

**FIGURE 1:**
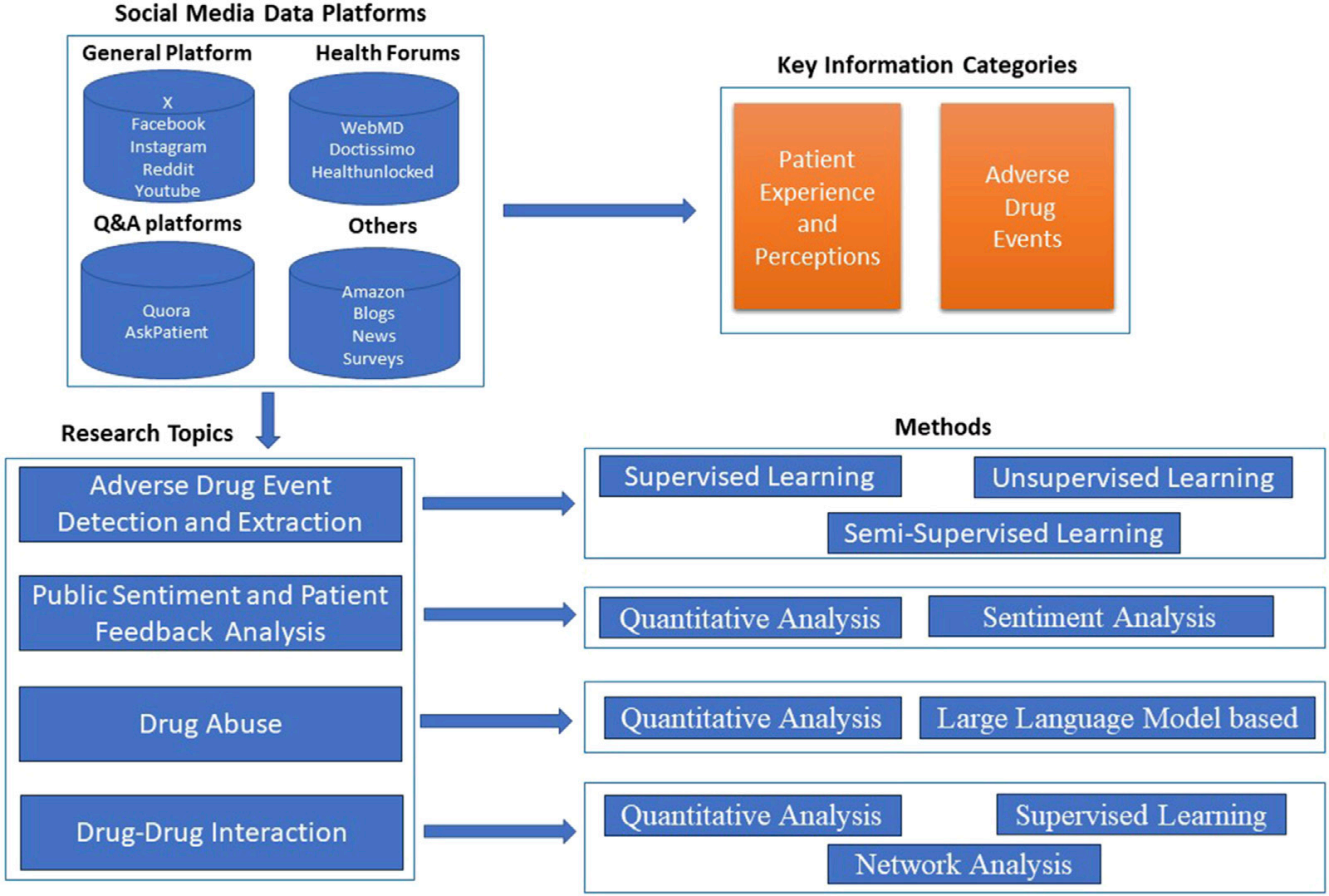
Overview of drug safety analysis with social media data.

Figure 1 provides an overview of the process for analyzing social media data to support pharmacovigilance. The process starts with data collection from various sources, including general social media platforms (e.g., X, Facebook, Instagram, Reddit, and YouTube), health-specific forums (such as WebMD and other medical forums), Q&A sites (such as Quora and Ask a Patient), and other online content (like Amazon reviews, blogs, news sites, and surveys). The information extracted from these sources is categorized primarily into Patient Experience and Perceptions and Adverse Drug Events. The extracted data undergoes further analysis in four areas:

1. Machine Learning methods for Adverse Drug Event Detection: This involves identifying and categorizing adverse drug events through methods such as supervised, semi-supervised, and unsupervised learning.
2. Public Sentiment and Patient Feedback Analysis: Techniques like quantitative analysis and sentiment analysis are applied to understand public opinion and patient feedback on drug safety.
3. Drug Abuse Monitoring: Quantitative analysis and advanced language models (large language model-based methods) are employed to monitor drug abuse trends based on user discussions.
4. Drug-Drug Interaction Monitoring: Network analysis and supervised learning are used to identify and assess potential interactions between different drugs.

Through these stages the process integrates various machine learning and analysis techniques to draw meaningful information that can enhance pharmacovigilance practices by capturing real-world patient experiences, monitoring safety signals, and improving our understanding of drug-related issues.

## Social media platforms utilized for pharmacovigilance

Social media platforms provide diverse online environments for sharing user-generated content which contains data useful for drug safety research. General platforms like X,^1^ Facebook,^2^ Instagram,^3^ Reddit,^4^ and YouTube^5^ offer broad audience engagement, while health-specific forums such as WebMD^6^ as well as Q&A platforms such as Quora^7^ and Ask a Patient^8^ focus on medical discussions. Additionally, other platforms such as the e-commerce site Amazon^9^ and various blogs have user reviews and discussions on drug products, enriching the data pool for drug safety analysis. Collectively, these social media platforms offer data that can facilitate a comprehensive understanding of patient experiences, medication effects, and healthcare trends.

Social media platforms differ in data scale, user demographics, content modality, and real-time accessibility—factors that shape their utilization in pharmacovigilance. X features high-frequency, short-text updates in real time, making it well-suited for detecting emerging safety signals, particularly among its predominantly young U.S. users. Facebook provides large-scale, globally diverse user demographics and facilitates lifestyle sharing, intergenerational interaction, and marketplace activity. Instagram contains visually rich content—photos and videos—primarily around themes like fashion, beauty, and travel, attracting a younger audience but offering limited textual data. Reddit hosts in-depth, community-driven discussions on topics such as technology and health through anonymous forums, though it lacks real-time immediacy. YouTube delivers both long- and short-form video content across educational and entertainment topics to a global, multi-generational audience, but pharmacovigilance efforts on the platform require resource-intensive video analysis. Health forums focus on structured, medically-oriented discussions within smaller, specialized communities. E-commerce platforms like Amazon provide valuable insight into over-the-counter medication safety through consumer reviews. Meanwhile, blogs and online surveys, though useful for exploring historical public opinion, are declining in relevance compared to more dynamic and interactive social media channels. These variations highlight the importance of tailoring pharmacovigilance strategies to each platform—leveraging the strengths of real-time surveillance on X or visual content on Instagram, while accounting for constraints in scalability, content richness, or demographic coverage.

Social media has evolved significantly since its early days in the 1970s, when online communication was limited to basic chat rooms and forums. In the 2000s, platforms such as Facebook, X (formerly Twitter), and Instagram emerged, enabling users to share experiences in real time. Over time, social media content has expanded from text-based posts to multimedia formats, including images, videos, and live interactions. Access for researchers has also changed—while many platforms initially offered open APIs for data analysis, recent restrictions have limited the availability of user-generated content, posing new challenges for social media-based research. Researchers are employing adaptive strategies such as web scraping, synthetic data generation, and collaborative research agreements with platforms to obtain unrestricted access to social media data for pharmacovigilance research, thereby mitigating the challenges posed by recent API limitations. Table 1 summarizes popular social media platforms and drug safety studies based on data extracted from these platforms.

**TABLE 1:** Data platform Review.

Category	Platform	Start year	Publication
General social media Platform	X	2006	[1–38]
	Facebook	2004	[1, 4, 19, 24–26, 28, 29, 38–47]
	Instagram	2010	[1, 4, 41, 48]
	Reddit	2005	[4, 16, 17, 41, 49–55]
	YouTube	2005	[1, 4, 41, 47, 56]
Online Health Forums	WebMD online health forum	1998	[40, 57, 58]
	Health forum in French (https://www.doctissimo.fr/)	N/A	[59]
	Health forum (Healthunlocked.com)	2010	[60]
Q&A platforms	Quora	2009	[61]
	Ask a Patient	2000	[62, 63]
Others	Amazon	1995	[64, 65]
	Blogs and News	N/A	[1, 24, 29, 43, 66–68]
	Online Surveys	N/A	[69–76]

X has emerged as a pivotal resource in pharmacovigilance, providing insight into public perceptions and experiences regarding medications. Researchers have effectively leveraged this platform to extract valuable information, including adverse drug events, user sentiments, and discussions on drug safety and efficacy. Through the application of analytical methods such as natural language processing, machine learning, and sentiment analysis, they have managed the challenges posed by extensive data volumes and noise in the user-generated content, thereby enhancing drug safety monitoring and public health strategies. For instance, Sule et al. [1] utilized X to combat the dissemination of COVID-19 misinformation from physicians, thereby contributing to the improvement of public health communications. Pathak and Catalan-Matamoros [2] explored X’s potential as an early warning system for drug safety signals, with the aim of strengthening pharmacovigilance practices. Hua et al. [8] investigated public perceptions of COVID-19-related medications using X, offering valuable insight for pharmacovigilance and policy-making decisions. Sharma et al. [11] conducted a scoping review utilizing sentiment analysis to comprehend patient experiences with pharmacotherapy. Rezaei et al. [12] applied deep learning techniques to detect adverse drug events on X, thereby enhancing the efficiency of safety concern identification. Lardon et al. [23] evaluated X as a supplementary data source for pharmacovigilance and compared it with traditional monitoring systems. Khademi et al. [32] developed a model by coupling topic modeling and transformer-based learning for the early detection of vaccine safety signals in X posts, and the detected adverse events were generally aligned with those reported in a spontaneous vaccine safety surveillance system, showcasing the potential of social media data for early detection of emerging vaccine safety issues. These examples underscore the versatility and value of X posts in complementing traditional pharmacovigilance methods, particularly during public health crises like the COVID-19 pandemic, and in understanding off-label medication use.

Facebook offers important user-generated data for pharmacovigilance, covering discussions, comments, and posts on medications, adverse drug reactions, and drug safety feelings. This data helps improve post-marketing drug safety surveillance. For example, Pierce et al. [26] explored the possibility of detecting drug adverse events in Facebook and X earlier than their reports in the FDA Adverse Event Reporting System (FAERS), demonstrating that social media platforms such as Facebook and X can be used for early detection of certain adverse events. Powell et al. [28] investigated the usefulness of Facebook in post-marketing drug safety surveillance by examining its effectiveness in capturing patient experiences and concerns on medications, indicating that Facebook provides data for the detection of early warning signs of potential drug safety. Coloma et al. [29] used Facebook in their evaluation of social media networks' contributions to drug safety surveillance, demonstrating the usefulness of the patient-reported information in pharmacovigilance. These studies collectively highlight the significant role of Facebook in pharmacovigilance, supporting better regulatory decisions and patient care.

Instagram, a popular social media platform owned by Facebook, Meta, allows users to share photos, videos, and engage with others through likes, comments, and direct messaging. For instance, Li et al. [48] focused on developing a machine learning approach for identifying and profiling illicit drug dealers on Instagram by analyzing posts and comments for hashtags and language patterns associated with illegal drug dealings. Through an examination of posts and comments, this study identified key information like hashtags and language patterns indicative of illegal drug activities. This research addresses concerns about drug sales on social media and suggests a mechanism that may help counteract such occurrences.

Reddit’s diverse communities offer a wealth of user-generated health data, making it a valuable platform for pharmacovigilance and public health research. For instance, Godinez et al. [51] analyzed Reddit discussions to gain an understanding of the experiences and concerns of individuals transitioning between HIV pre-exposure prophylaxis medications, thereby informing healthcare policies. Guo et al. [50] utilized the Reddit platform for real-time tracking and analysis of COVID-19 symptoms, facilitating early detection and response to the pandemic. Furthermore, Szczypka et al. [55] explored Reddit discussions on Delta-8-tetrahydrocannabinol, shedding light on public perceptions and potential health risks associated with its use. Lastly, Sharp et al. [53] conducted a comprehensive analysis of 11 years of Reddit posts related to dietary supplements among military personnel, providing valuable data on safety, efficacy, and usage patterns within this specific population. These examples collectively underscore the significant role of Reddit in enhancing our understanding of drug safety and informing public health strategies.

YouTube, being a widely utilized video-sharing platform, provides a rich source of information on medication usage and health-related topics. For example, Hansen et al. [56] assessed the accuracy of safety information conveyed in YouTube videos about medication usage during pregnancy. Their aim was to ensure that pregnant women have access to reliable and evidence-based guidance to facilitate informed decision-making for their well-being.

Research on drug safety is increasingly leveraging patient-generated data from health-specific forums and online consultation platforms, such as WebMD. These studies utilize quantitative analysis and natural language processing techniques to extract information directly from patient reviews and discussions, providing a unique perspective on medication adherence, adverse drug reactions, and patient satisfaction. By comparing patient-reported reasons for medication changes against formal adverse event reporting systems and assessing the cognitive levels and intervention preferences of patients with hypertriglyceridemia, these research efforts enhance our understanding of real-world medication experiences and safety concerns, ultimately contributing to the improvement of pharmacovigilance practices.

In addition to WebMD, numerous health forums cater to different languages and focus on various topics. For instance, Abdellaoui et al. [77] used a topic modeling approach to identify instances of noncompliance to drug treatment in patient forum posts. Similarly, Karapetiantz et al. [78] found a discrepancy between personal experiences and negative opinions with the human papillomavirus vaccine in web forums. They also found that descriptions of adverse drug reactions are less detailed in forums compared to the French Pharmacovigilance Database,^10^ however, forums provide more unexpected reactions [79]. These examples illustrate the potential of health-specific forums and online consultation platforms in enhancing our understanding of drug safety and improving pharmacovigilance practices. They emphasize the importance of considering patient experiences and perspectives in different languages and contexts. Such an approach not only enriches available data for analysis but also ensures a more comprehensive and inclusive understanding of drug safety.

Q&A platforms like Quora and patient feedback websites such as^8^ play a crucial role in drug safety research by providing a space for patients to share their experiences, ask questions about medications, and discuss their treatment preferences. For instance, Xu et al. [61] analyzed the discussions about COVID-19 vaccine clinical trials on Quora, providing insight into public sentiment and common questions about the trials, which can inform future communication strategies and patient education efforts. Similarly, Song et al. [62] conducted a social media listening infosurveillance study to evaluate the needs and experiences of patients with hypertriglyceridemia. They identified common concerns and preferences that can be utilized to guide the development of patient-centered care strategies for this population. Moreover, Duh et al. [63] explored whether social media data could aid in the early detection of drug-related adverse events. They found that patient discussions on platforms like^8^ can provide early warning signs of potential adverse events, thereby enhancing the timeliness and effectiveness of pharmacovigilance efforts. These examples underscore the potential of Q&A platforms and patient feedback websites in drug safety research, emphasizing the importance of incorporating patient voices in the study to improve drug safety practices.

E-commerce platforms like Amazon serve as valuable repositories of consumer-generated data for drug safety research. By examining user reviews, ratings, and Q&A sections, researchers can gain insight into the real-world performance and safety of over-the-counter medications and health products. For instance, Adams et al. [64] used an automated method to uncover safety and efficacy issues related to joint and muscle pain treatments from Amazon reviews. Similarly, Gartland et al. [65] focused on creating crowdsourced training datasets for pharmacovigilance intelligent automation, illustrating the potential of platforms like Amazon Turk to provide valuable data for training machine learning models in pharmacovigilance studies. These examples highlight the significance of e-commerce platforms in drug safety research, enhancing our understanding of medication safety and efficacy in real-world contexts.

In the past, news articles and blog posts were frequently used as primary data sources for drug safety research. However, their utilization has witnessed a decline in recent years. Despite the shift from blogs to real time social media platforms, traditional news and blog data can still yield valuable insights for drug safety research. For example, de Vries et al. [66] analyzed a series of healthcare provider communications in newspaper articles spanning from 2001 to 2015 in the Netherlands, demonstrating the value of such media in drug safety research. Similarly, Matsuda et al. [68] analyzed patient narratives sourced from disease-specific blogs, a component of the TOBYO database,^11^ revealing valuable real-world medication experiences and emphasizing the potential of such platforms in bolstering drug safety research and pharmacovigilance efforts. These examples emphasize the significant role of news and blog data in understanding public perceptions and experiences related to drug safety.

Online surveys are valuable tools in drug safety research, providing a platform for collecting large-scale patient-generated data. For instance, Grundmann et al. [70] used an online survey to investigate patterns of Kratom use and its health impact in the US. Similarly, Wysota et al. [75] and Nguyen et al. [76] utilized online surveys to understand consumers’ knowledge, perceptions, and usage of cannabidiol products. The widespread adoption of online surveys signifies their pivotal role in advancing drug safety research by providing a robust platform for data collection and analysis.

## Information extracted from social media platforms

In the realm of pharmacovigilance, social media has emerged as a pivotal source of information, offering unprecedented access to a wealth of data regarding patient experiences, perceptions, and discussions related to medication use and safety. This section delves into the various ways social media data can be harnessed to enhance our understanding of patient experiences, detect and analyze adverse drug events, assess vaccine efficacy and safety, and combat the spread of misinformation. It highlights the importance of leveraging social media platforms to inform healthcare practices, improve pharmacovigilance efforts, and address the challenges posed by misinformation, ultimately aiming to enhance public health and safety. Table 2 shows the categories of key information extracted from social media.

**TABLE 2:** Extracted key information categories from social media data.

Key information Category	Key information	Reference
Patient Experience and Perceptions	patient experience of adverse events for Discontinuing Statin Therapy	[40]
	Patient experience of adverse events for medication change	[57]
	Patient experience of fenbendazole safety and efficacy	[42]
	Negative opinions and personal experience with HPV vaccine	[78]
	Patient reported Symptoms	[50]
	Patient Experiences with Dabigatran	[80]
	consumer perceptions and attitudes on cannabis products	[76]
Adverse Drug Events	Adverse Drug Events in X posts	[2, 3, 5, 10, 14, 22, 34, 81–83]
	Adverse Drug Events in Forums	[59, 72, 84, 85]

### Patient experiences and perceptions

Social media platforms have become invaluable for capturing patient experiences and perceptions regarding medication use, benefits, and safety. Golder et al. [40] and Micale et al. [57] examined online discussions and personal narratives, uncovering reasons behind medication changes, such as adverse events experienced by patients on statin therapies and other treatments, along with factors influencing their satisfaction and concerns. Yamaguchi et al. [42] explored the impact of social media information on self-medication choices, focusing on fenbendazole, and highlighting how such information can lead to adverse events, emphasizing the need for effective communication strategies and vigilant monitoring. In a similar vein, Karapetiantz et al. [78] analyzed HPV vaccine discussions in online forums, identifying a gap between personal experiences and negative public opinions, which contributes to vaccine hesitancy and signals the need for targeted interventions. Guo et al. [50] further illustrated how social media enhances traditional pharmacovigilance by offering a deeper understanding of real-world drug safety through patient-reported symptoms. Vaughan et al. [80] documented patient experiences with dabigatran, offering insight into patient satisfaction, concerns, and adverse effects that stress the importance of understanding patient perspectives. Additionally, Nguyen et al. [76] highlighted consumer perceptions and attitudes toward cannabis products, demonstrating the influence of social media in shaping public opinion and highlighting concerns related to the use and safety of such products. Collectively, these studies showcase social media’s role in strengthening pharmacovigilance by amplifying patient voices and informing healthcare practices that align with patient needs and real-world experiences.

### Adverse drug events:

Social media platforms, especially X, are increasingly being used to detect adverse drug events. Pathak et al. [2] and Litvinova et al. [3] demonstrated the potential of X posts as early indicators of safety signals, with Pathak focusing on initial detection and Litvinova refining methods for accurate adverse drug event identification. Yu et al. [5] developed a natural language processing model to analyze X posts for adverse drug events, while Magge et al. [10] used deep learning to extract adverse drug events from the platform. The Web-RADR project, as reported by Gattepaille et al. [14], applied machine learning to identify adverse drug events on X, establishing a benchmark dataset for adverse event recognition. Masino et al. [22] enhanced automated adverse drug event detection using convolutional neural networks, and Fisher et al. [34] introduced a scalable machine learning framework for identifying drug-related harms on social media. Further advancements in ADE detection on social platforms were achieved by Botsis et al. [81], Dong et al. [82], and Zhang et al. [83] through the application of text mining and BERT-based language models, enhancing post-marketing surveillance and pharmacovigilance practices.

Web forums also serve as valuable source for monitoring adverse drug events. Roche et al. [59] extracted adverse drug events from Doctissimo, a medical forum where users report side effects and allergic reactions. Bulcock et al. [72] highlighted HealthUnlocked, a health discussion forum, as a platform for detecting emerging side effects. Audeh et al. [84] explored French web forums where users share serious adverse drug events, and Karapetiantz et al. [85] analyzed 23 health forums using the V4M Scraper tool to identify adverse drug events, drug interactions, and safety concerns. Together, these studies showcased the potential of social media and web forums as real-world sources of adverse drug event data, enriching drug safety research and enabling faster responses to public safety concerns.

## Methods for utilizing social media data

The integration of social media data into drug safety monitoring has significantly advanced the detection of adverse drug events, analysis of public sentiment, monitoring of drug abuse, and identification of drug-drug interactions. Social media platforms, with their vast and diverse user-generated content, offer a unique perspective on real-world patient experiences and public perceptions of medications.

In adverse drug events detection, both supervised and unsupervised learning methods, including models like BERT and co-clustering algorithms, are applied to extract and analyze adverse events from social media posts. For public sentiment analysis, cutting-edge sentiment analysis models are being used to assess patient feedback and identify potential safety signals. Meanwhile, drug abuse monitoring employs large language models and quantitative analysis to detect trends in misuse, addiction, and overdose incidents. The identification of drug-drug interactions is enhanced through network analysis and supervised learning techniques.

These methods are reshaping pharmacovigilance, enabling faster identification of safety concerns, a deeper understanding of patient experiences, and more effective monitoring of public health issues. Table 3 summarizes the various methods and their applications in utilizing social media data to advance drug safety studies.

**TABLE 3:** Methods for analyzing adverse drug events, public sentiment, drug abuse, and drug-drug interactions using social media data.

Topic Category	Method Category	Details	Advantage	Limitation	Reference
Machine Learning methods for Adverse Drug Event Detection	Supervised Learning	BERT-based model to extract adverse drug events from X posts	High accuracy on X posts	Requires labeled data for effective training	[82]
		natural language processing methods to detect adverse drug events from clinical text	High accuracy in structured clinical environments	Limited generalizability to non-clinical text and informal language	[86]
		Pipeline to extract and normalize adverse drug events	Detect adverse drug events in X posts	Limited to X posts data	[10]
		adverse drug event detection across platforms	High detection accuracy	Limited generalizability to diverse social media platforms	[12]
	Unsupervised Learning	Co-clustering for adverse drug event detection	Detects adverse drug event signals by clustering related data without predefined labels	Validation limited to COVID-19 related data	[87]
		Transformer-based adverse drug event detection	High sensitivity in capturing emerging adverse drug events	Limited to specific drugs and topics in the training set	[49]
		CNN model for adverse drug event detection	Detects adverse drug events without predefined labels	Limited in capturing complex adverse drug events	[22]
	Semi-supervised Learning	Word embeddings-based lexical network for adverse drug events detection	Severity scoring to assess different adverse drug events	Data quality, noise, and bias exist in social media data	[54]
Public Sentiment and Patient Feedback Analysis	Quantitative Analysis	Direct patient feedback analysis through quantitative methods	Provides insight into patient perspectives	Limited by the accuracy of self-reported data	[57]
	Sentiment Analysis	WC-CNN model for sentiment analysis	Detects safety signals by examining user behavior over time	Limited to the Levothyrox case in France	[59]
		Sentiment analysis of public perception of specific drugs for COVID-19 treatment	Captures public perceptions on certain drugs	Limited to specific drugs and COVID-19 context	[39]
		VADER model for sentiment analysis on cannabidiol use for various conditions	Provides insight into public perception of cannabidiol use	Manual labeling needed for classifier training	[9]
Drug Abuse	Large Language Model based	GPT-3 for generating a lexicon for drug abuse detection in social media	Captures slang and misspellings for drug abuse detection	Limited to 98 drugs with limited evaluation	[88]
	Quantitative Analysis	Frequency analysis of opioid misuse, abuse, addiction, overdose, and death	Real-time tracking and insight into opioid abuse trends	Limited to opioid-related data	[36]
		Statistical analysis with data quality metrics and topic modeling for drug abuse content	Structured framework for analyzing drug abuse-related content	Limited social media platforms and limited generalizability	[89]
		NER for adverse drug event identification; PRR for potential adverse drug event detection; topic modeling to identify themes	Comprehensive analysis and real-time monitoring using social media data	Limited to methylphenidate-related posts between 2007 and 2016 only	[90]
		Quantitative analysis of annotated posts for drug abuse	Supervised classification model to detect drug abuse signals in X posts	Requires manually annotated 6,400 X posts containing drug abuse signals	[91]
Drug-Drug Interactions	Quantitative Analysis	Frequency and rates for posts related to Drug-Drug Interactions are analyzed	Manually reviewed and evaluated by two blinded investigators	Limited data and limited manual validation	[92]
	Supervised Learning	Drug-Drug Interactions detection in social media data	Extracts related Drug-Drug Interactions information from social media platforms	Requires an annotated Drug-Drug Interactions corpus	[93]
	Network Analysis	Co-occurrence network for users based on potential Drug-Drug Interactions	Detects relationships and patterns for emerging Drug-Drug Interactions through network analysis	Limited to Instagram platform with privacy and ethical concerns	[94]

### Machine learning methods for adverse drug event detection

One of the most active areas of drug safety research is the detection and extraction of adverse drug events from social media posts. Various machine learning approaches—supervised, unsupervised, and semi-supervised—are employed, each with unique strengths and limitations.

Supervised learning approaches, such as those utilizing BERT-based models, achieve high accuracy in extracting adverse drug events but are heavily reliant on large, labeled datasets. This requirement makes them resource-intensive and less adaptable to diverse social media platforms. For example, BERT-based models have demonstrated exceptional performance in extracting adverse drug events from X posts, but their effectiveness hinges on the availability of labeled data [82]. Natural language processing techniques have also been used to detect adverse drug events in clinical documents, achieving good results within structured data but facing challenges when applied to informal or unstructured social media text [86]. Magge et al. [10] used deep learning methods in their DeepADEMiner pipeline to extract and normalize adverse drug event mentions on X posts. Despite its success on specific platforms like X, such models often struggle to generalize across the diverse and evolving landscape of social media environments [12].

Unsupervised learning methods, such as co-clustering, allow adverse drug event detection by grouping related data without requiring predefined labels. These methods have been particularly useful in monitoring adverse drug events associated with COVID-19, though their validation remains largely confined to this context [87]. Transformer-based [49] and CNN-based [22] models enhance adverse drug event detection sensitivity and capture emerging adverse drug events without relying on labeled data. However, these approaches are often constrained by the drugs and topics present in the training set, making them less adaptable to new or evolving drug discussions.

Semi-supervised learning techniques combine the strengths of supervised and unsupervised techniques, using a smaller labeled dataset alongside a larger pool of unlabeled data. This approach strikes a balance between accuracy and flexibility, although it continues to face challenges with scalability and data quality. For instance, word embeddings-based lexical networks have been used to introduce severity scoring for assessing adverse drug events, addressing the inherent issues with data quality, noise, and bias in social media data [54].

In summary, while advancements in supervised, unsupervised, and semi-supervised learning techniques have significantly improved ADE detection from social media, each method has inherent trade-offs. Future research must focus on enhancing generalizability, scalability, and robustness to fully leverage social media data for pharmacovigilance.

### Public sentiment and patient feedback analysis

Social media platforms provide unique patient perspectives and public sentiment regarding medications, complementing traditional pharmacovigilance methods. Quantitative analysis methods and sentiment analysis techniques contribute to understanding patient experiences and detecting drug safety signals, though challenges with data quality and generalization persist.

Quantitative analysis methods, often involving manual review of patient posts, offer valuable information on patient attitudes and experiences. However, the accuracy and reliability of self-reported data remain significant limitations [57]. Despite these constraints, such methods are instrumental in capturing nuanced patient feedback and identifying trends in medication use and side effects.

Sentiment analysis techniques are frequently used to track public perceptions of medications and detect emerging drug safety signals. Advanced models like WC-CNN have been employed to monitor sentiment trends linked to specific drugs, such as Levothyrox in France. While effective in capturing localized sentiment, these models often struggle to generalize across different drugs or regions [59].

Studies have also employed sentiment analysis to understand public perceptions of drugs for specific conditions. For instance, sentiment analysis models like VADER have been applied to assess public attitudes toward COVID-19 treatments [39] and the use of cannabidiol for various health issues [9]. While VADER provides valuable public sentiment, its reliance on manual labeling for classifier training presents scalability challenges, particularly when dealing with large, diverse datasets.

Together, quantitative and sentiment analysis methods enrich drug safety assessments, offering a more comprehensive understanding of medication safety and public attitudes. However, ongoing efforts to address issues with data quality, scalability, and model generalization are essential for maximizing their potential in pharmacovigilance.

### Drug abuse

The rise of drug abuse, particularly opioid misuse, has driven significant efforts to monitor drug abuse trends using social media data. By analyzing user-generated content, researchers aim to enable early detection of abuse, addiction, overdose, and other drug-related issues.

Large language models like GPT-3 have been used to generate lexicons for drug abuse detection, capturing slang, misspellings, and colloquialisms frequently used in drug-related discussions. While these lexicons offer a valuable starting point, they typically focus on a narrow range of drugs and often lack comprehensive evaluation, limiting their broader applicability [88].

Quantitative analysis methods, such as frequency analysis, provide real-time tracking of opioid misuse, abuse, addiction, overdose, and related deaths. These methods help identify emerging trends and patterns in drug abuse [36]. More structured approaches, incorporating data quality matrices and topic modeling, provide frameworks for analyzing drug abuse content across social media platforms. However, the diversity and variability of these platforms pose challenges to generalizability and cross-platform applicability [89].

Techniques such as named entity recognition combined with proportional reporting ratios enables more targeted analysis by identifying specific drugs and associated adverse events. For instance, studies have focused on methylphenidate-related social media posts from 2007 to 2016, highlighting the potential of such methods for longitudinal drug abuse research [90].

Supervised learning models further enhance drug abuse monitoring. Sarker et al. [91] developed a supervised classification model trained on 6,400 manually annotated X posts to identify drug abuse-related content. This approach highlights the importance of labeled data in improving the precision and reliability of drug abuse trend analysis on social media.

By combining advanced natural language processing techniques with structured analytical methods, researchers are gaining a deeper understanding of drug abuse trends. However, challenges related to data quality, platform diversity, and scalability remain areas for future improvement.

### Drug-drug interaction

Monitoring drug-drug interactions using social media data is an emerging trend in drug safety evaluation. Social media provides the advantage of real-time, user-generated data, allowing for quick identification of potential drug-drug interactions across diverse populations. However, this approach faces challenges, including the need for context-rich analysis, issues with data quality, and privacy concerns on certain platforms. Traditionally, drug-drug interactions detection relied on structured data, but social media offers a dynamic and expansive source of information.

Quantitative approaches analyze the frequency and prevalence of drug-drug interactions related posts, which are then manually reviewed by blinded investigators. While informative, these methods are often limited by data availability and require labor-intensive validation processes [92]. Supervised learning models address these limitations by leveraging annotated drug-drug interactions corpora to extract relevant interaction information from user posts, enhancing detection efficiency [93].

Network analysis has also been applied to study potential drug-drug interactions by constructing co-occurrence networks based on user mentions of interacting drugs. This method facilitates the identification of emerging drug-drug interaction patterns and relationships. However, its application is sometimes limited by platform-specific privacy and ethical concerns, especially on platforms like on Instagram [94].

In conclusion, leveraging social media for monitoring drug-drug interactions presents a promising complement to traditional methods, offering real-time information and access to diverse patient populations. While challenges such as data quality, contextual interpretation, and privacy concerns remain, advancements in quantitative analysis, supervised learning models, and network analysis demonstrate the potential of this approach. With continued refinement and ethical considerations, social media could become a vital tool in enhancing drug safety and public health.

## Discussion

Integrating social media data into drug safety research offers valuable insight, but presents several challenges that must be addressed to ensure reliable and actionable findings.

### Data reliability and quality

Social media data is often unstructured and informal, complicating the identification of credible adverse drug event reports amidst speculative or inaccurate posts. The variability and frequent lack of contextual information in social media content further hinder interpretation, reducing the reliability of findings. Addressing these issues requires rigorous screening, validation, and contextual analysis. Moreover, the lack of demographic and medical context from social media users makes it challenging to generalize findings across populations. Comparing and pooling data across social media platforms is challenging due to differences in user demographics, content formats, and engagement patterns. Privacy regulations further restrict access to user profiles, making it difficult to analyze demographic information comprehensively. Additionally, a key limitation of current studies is the inability to fully verify whether social media users are real individuals or automated accounts. While many platforms have implemented stricter user verification measures, restricted access to demographic and account data remains a significant challenge for researchers seeking to ensure the authenticity and representativeness of social media-derived insights.

Social media platforms often restrict access to demographic and account information, posing challenges in verifying user identities. AI tools can help mitigate this by detecting bot-like behavior, such as rapid posting patterns and generic language. However, the rise of advanced language models introduces new concerns, as they can generate large volumes of content that may be difficult to distinguish from genuine user experiences. To enhance data reliability, preprocessing need to include methods for filtering posts from suspicious or abnormal accounts, ensuring that subsequent analysis focuses on real-world health experiences shared by actual users.

Data reliability is a key concern in social media-based pharmacovigilance. For data preprocessing, inclusion criteria focus on posts that explicitly mention drugs, adverse drug events, or health experiences. To ensure quality, irrelevant content, spam, and non-English posts are removed. Additionally, posts from suspicious or abnormal user accounts are filtered out to enhance reliability and prioritize real user experiences. While confirming individual accounts as real people remains a challenge, these preprocessing steps help improve data integrity and the robustness of findings.

### Ethical and privacy concerns

The use of personal social media data in drug safety research introduces significant ethical and privacy challenges. Researchers must comply with privacy laws and adhere to ethical standards to mitigate risks such as discrimination or stigmatization. Implementing robust privacy protections, anonymization techniques, and transparent data usage practices is essential to maintain public trust and protecting users.

### Misinformation and bias

Social media is prone to misinformation and biases that can hinder accurate adverse drug event detection. Distinguishing genuine patient experiences from misleading or biased content requires careful fact-checking and verification to ensure data reliability. Misinformation on social media can distort perceptions of drug safety, emphasizing the need for robust methods to counteract its influence. A key research gap is the need for a deeper exploration of strategies to mitigate the impact of misinformation and bias, as these factors influence all reported findings and approaches discussed in this review. Future research could focus on developing and evaluating advanced AI-driven algorithms for detecting and filtering misinformation. Additionally, platform regulations, combined with multi-source verification mechanisms, could play a crucial role in limiting the spread of biased or inaccurate content while still addressing user privacy concerns. A more systematic investigation into these mitigation strategies would strengthen the reliability of social media-based research findings.

### Text mining and modeling challenges

Text mining models face limitations when processing the informal language common on social media, such as slang, abbreviations, and non-standard terms. This issue can reduce the accuracy of automated adverse drug event detection. To address these issues, researchers need to improve text mining models and standardize data validation methods. Combining social media data with traditional pharmacovigilance methods provides a more comprehensive understanding of drug safety.

### Regulatory frameworks

The regulatory landscape for incorporating social media data into pharmacovigilance is still evolving. Clear and standardized guidelines are needed to govern ethical data use and alignment with established pharmacovigilance practices. Such frameworks are crucial for maintaining public trust and facilitating the effective use of social media in drug safety research.

## Conclusion

The integration of social media data into drug safety research offers promising opportunities to enhance pharmacovigilance. Social media’s vast, real-time data can complement traditional methods, enabling early detection of adverse drug events, monitoring public sentiment, and capturing patient experiences on medication risks and benefits. However, unlocking the full potential of social media in drug safety research requires addressing significant methodological, ethical, and regulatory challenges.

This review highlights the need for rigorous methodologies, including advanced data validation, standardized data processing protocols, and specialized text mining models tailored to the unique characteristics of social media content. Ethical considerations, such as privacy protection and transparent practices, are essential to maintaining public trust. Additionally, as social media evolves, developing a strong regulatory framework to govern the use of social media data in pharmacovigilance is important.

Future research should focus on developing real-time monitoring systems that can utilize social media for the early detection of drug safety concerns. Hybrid models that integrate social media data with traditional pharmacovigilance data hold promise for the potential to improve the accuracy, speed, and scope of safety signal detection. Standardizing data collection and analytical methodologies across studies will enhance consistency and reliability, enabling more actionable findings. Addressing cultural and linguistic diversity in social media content will also be essential to adapt these approaches to global pharmacovigilance efforts.

Collaboration among academia, industry, regulatory agencies, and social media platforms will play a key role in advancing these goals. Such partnerships can foster resource sharing, methodological alignment, and innovation in drug safety monitoring. Regulatory agencies can provide guidance on ethical and privacy standards, while social media platforms can facilitate data access and support anonymization efforts. These collaborative efforts will help establish a robust and responsive pharmacovigilance ecosystem, enhancing post-market surveillance and protecting public health worldwide. By integrating social media data with established pharmacovigilance practices, researchers and healthcare providers can improve post-marketing drug safety monitoring, enhance public health outcomes, and build a collaborative ecosystem that benefits patients and society.

## Footnotes

1
https://x.com/

2
https://www.facebook.com/

3
https://www.instagram.com/

4
https://www.reddit.com/

5
https://www.youtube.com/

6
https://www.webmd.com/

7
https://www.quora.com/

8
https://askapatient.com

9
https://www.amazon.com/

10
https://ansm.sante.fr/page/enhanced-surveillance-of-medicines

11
https://www.tobyo.jp/
